# Analysis of Vibration Characteristics of Spatial Non-Uniform Tensioned Thin-Film Structures Based on the Absolute Nodal Coordinate Formulation

**DOI:** 10.3390/mi15091147

**Published:** 2024-09-12

**Authors:** Peng Sun, Jin Huang, Jiaying Zhang, Fanbo Meng, Pengbing Zhao

**Affiliations:** State Key Laboratory of Electromechanical Integrated Manufacturing of High-Performance Electronic Equipments, Xidian University, No. 2 South Taibai Road, Xi’an 710071, China; 20041110177@stu.xidian.edu.cn (P.S.); 19041110403@stu.xidian.edu.cn (J.Z.); pbzhao@xidian.edu.cn (P.Z.)

**Keywords:** Absolute Nodal Coordinate Formulation (ANCF), non-uniform elements, thin-film structure, wrinkle deformation, vibration characteristics

## Abstract

Due to their lightweight characteristics, spatial thin-film structures can generate vibrations far exceeding their film thickness when subjected to external loads, which has become a key factor limiting their performance. This study examines the vibration characteristics of tensioned membrane structures with non-uniform elements subjected to impacts in air, leveraging the Absolute Nodal Coordinate Formulation (ANCF). This model takes into account the wrinkling deformation of thin films under pre-tension and incorporates it into the dynamic equation derived using the absolute node coordinate method. A detailed discussion was conducted on the influence of non-uniform elements, situated at different locations and side lengths, on the vibration characteristics of the thin film. The analytical results obtained from the vibration model were compared with the experimental results, validating the effectiveness of the vibration model. This provides a theoretical foundation for the subsequent vibration control of thin films.

## 1. Introduction

Large-scale thin-film structures have received widespread international attention due to their lightweight and foldable characteristics [1,2,3,4]. The introduction of non-uniform structures into these thin-film structures has generated significant interest, such as in thin-film antenna structures and solar sail structures equipped with actuators in aerospace engineering [5,6,7]. Thin films are highly susceptible to vibrations far exceeding their thickness when subjected to impact loads and can develop wrinkles under preloads. The introduction of non-uniform structures not only affects the static characteristics of the thin film, such as stress distribution and wrinkling behavior, but also alters its vibration characteristics under impact [8,9,10]. Therefore, studying the vibration characteristics of non-uniform thin-film structures holds profound theoretical value for designing high-performance spatial thin-film structures.

Currently, scholars have conducted extensive research on the vibration characteristics of thin-film structures [11,12,13]. However, the current research is limited to the free vibration of uniform thin-film structures under no external force, and relatively little attention has been paid to the impact vibration characteristics of non-uniform tensioned thin-film structures. Li established a free vibration model for rectangular thin films considering the added mass of air based on the stress superposition method and discussed several characteristics related to the fundamental frequency and mode shapes of rectangular thin films [14]. Liu combined stability theory to establish a wrinkling prediction model for thin films under tension and further developed a dynamic model considering thin film wrinkling, describing the impact of wrinkling on the vibration characteristics of the thin-film structures [15]. Li proposed an eigenvalue method capable of analyzing the vibration characteristics of wrinkled and deformed thin films, based on the stress distribution pattern in the wrinkled region, and a dynamic model for rectangular sheared wrinkled thin films was established. The research results showed that the vibration frequency of the thin-film structure increased significantly with the vibration of wave peaks [16]. Liu studied the coupling effect between wrinkling and vibration and found that wrinkling has a significant impact on the dynamics of thin-film structures under asymmetric tensile loads [17]. Lu established a nonlinear dynamic model for tensioned thin-film antennas during motion, describing the influence of the rigid–flexible coupling effect on structural stiffness and damping characteristics [18]. Wang studied the free transverse vibration of annular wrinkled thin films, established a dimensionless Hamiltonian equation of motion for annular wrinkled thin films, and solved it using the finite difference method to obtain their vibration frequencies and mode shapes [19]. Fan established an analytical model for post-buckling membrane vibrations on a flexible substrate and systematically studied the analytical prediction of the system’s natural frequency and mode shape [20]. Wang established a linear self-vibration equation for flexible substrate thin films and studied the effects of substrate stiffness and damping on the natural frequency and vibration mode of the substrate thin films [21].

Previous studies have primarily focused on analyzing the vibration characteristics of the thin film itself, neglecting the vibration of the cables during the tensioning process. Therefore, they could not accurately capture the vibration characteristics of the entire thin-film structure. In the ANCF, similar to the classical finite element method, the object is discretized using finite elements, but the gradients of absolute position vectors are used as node coordinates instead of rotations. This description allows for a constant mass matrix with zero Coriolis and centrifugal forces. Conversely, the stiffness matrix is a highly nonlinear function of the element node coordinates [22,23].

Skrinjar used the absolute node coordinate method to model the prestressed rigid–flexible composite body in rigid–flexible assembly and studied the influence of different parameters on its dynamic response [24]. Ren proposed an adaptive triangular element based on the absolute node coordinate method, established a fine theory of thin plates and thin films, and analyzed the dynamic response of thin plates and the wrinkling and buckling behavior of thin films through an adaptive algorithm based on the stress state [25]. Wang conducted an unfolding simulation of foldable thin-film structures based on the absolute node coordinate method and integrated the nonlinear unfolding of foldable thin films into multi-body systems using the principle of virtual work [26]. Liu established a thin-film model using reduced plate elements and studied the wrinkling and buckling behavior of tensioned thin films and the dynamic response of rotating solar sails [27]. Yuan conducted a dynamic modeling of a folded-space thin-film structure subjected to contact impact during the unfolding process and discretized the thin film using triangular elements of the absolute node coordinate method to determine the stress–strain relationship of the thin film [28]. Zhao et al. conducted a modal analysis on rotating thin plates using the absolute nodal method and studied the effect of rotational angular velocity on natural frequency [29]. Wang proposed a multi-node flat plate element based on the absolute node coordinate method to calculate elastic and inertial functions and defined the displacement field using the bidirectional univariate Lagrange interpolation method, verifying the effectiveness of the ANCF element in static and dynamic analyses [30]. Olshevskiy proposed a fully parameterized ANCF flat plate element using only the transverse slope to simulate large displacements and rotations of thick plates [31]. Shababa defined a displacement rotation field based on the ANCF and used a first-order difference method to avoid the redundancy problem encountered in formulas with large rotation vectors [32]. Mikkola defined the relationship between B-spline surface control points and ANCF positions and gradient vectors through linear transformation, resulting in a unique displacement and rotation field that transforms B-spline surfaces into ANCF thin-plate finite elements [33]. Pappalardo developed a rational absolute nodal coordinate formula for thin-plate elements, which can be used for precise geometric modeling and the analysis of flexible continuum structures with complex geometries [34]. Grossi studied the high-frequency modes generated by the application of ANCF in multi-body systems and effectively solved rigid differentiation and memory groups by filtering and suppressing ANCF high-frequency modes, thereby reducing the negative impact of high-frequency modes on computational efficiency [35]. Pappalardo and Zhang developed a new fully parameterized ANCF triangular plate/shell element that defines the inertia and elasticity of the structure through element transformation between volume and Cartesian parameterization [36].

To obtain the dynamic characteristics of a multibody wrinkle thin-film system with cables, thin films, and non-uniform elements under impact, this study utilized reduced beam and thin-film elements to establish a multibody thin-film-structure dynamic model. Based on the non-uniform thin-film-wrinkling prediction model, the out-of-plane wrinkling deformation of thin films was obtained and introduced as an initial boundary into the dynamic model. Impact vibration analysis was performed on the tensioned wrinkled thin films with cables, and the vibration curves at the center of the thin film and the non-uniform element were extracted and compared with the experimental results to validate the effectiveness of the dynamic model.

## 2. ANCF Elements with Insufficient Gradients

### 2.1. Reducing Beam Elements

**Figure 1:**
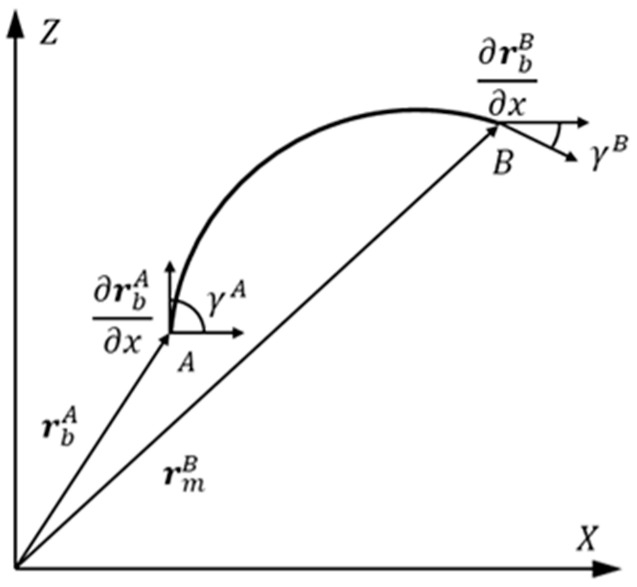
Schematic diagram of reduced beam elements.

The full parameter beam element can be used to describe the deformation of the cross-section of Timoshenko beams. The cable structure in this article can be regarded as a Euler–Bernoulli beam, and the deformation of the cross-section is usually not considered. Therefore, a reduced beam element can be used for description. In the membrane structure under impact, the cables are mainly subjected to tensile deformation along their axis and vibration deformation along the normal direction of the membrane. Reduced beam elements can be used to model the cables. Divide the lasso into *m* elements, each containing two nodes and 12 degrees of freedom. Take any element within the lasso and establish a global coordinate system, as shown in Figure 1. Among them, $r_{b}^{A}$ and $r_{b}^{B}$ are the loss paths of node *A* and node *B* in the cable relative to the global coordinate origin *O*, respectively, γ is the inclination angle of the tangent at any point within the unit relative to the X-axis and γ. The gradient with respect to the position coordinate, denoted as ∂r/∂x, should satisfy the following relationship:

(1) $$\begin{array}{l} \cos\gamma =\frac{\partial r_{x}/\partial x}{\sqrt{{\left(\partial r_{x}/\partial x\right)}^{2}+{\left(\partial r_{y}/\partial x\right)}^{2}}},\\ \sin\gamma =\frac{\partial r_{y}/\partial x}{\sqrt{{\left(\partial r_{x}/\partial x\right)}^{2}+{\left(\partial r_{y}/\partial x\right)}^{2}}}. \end{array}$$

The shape function and node vector coordinates of the reduced beam element are defined as follows:

(2) $$r_{b}=S^{b}\left(x\right)e_{b},$$

where $S^{b}\left(x\right)$ is a reduced beam element form function matrix represented by spatial coordinate *x*, which can be expressed as follows:

(3) $$S^{b}\left(x\right)=\left[\begin{array}{cccc} S_{1}^{b}I_{2} & S_{2}^{b}I_{2} & S_{3}^{b}I_{2} & S_{4}^{b}I_{2} \end{array}\right],$$

where the elements of the shape function can be expressed as follows:

(4) $$\begin{array}{cc} S_{1}^{b}=1-\xi_{b}^{2}+2\xi_{b}^{3} & S_{2}^{b}=L\left(\xi_{b}-2\xi_{b}^{2}+\xi_{b}^{3}\right)\\ S_{3}^{b}=3\xi_{b}^{2}-2\xi_{b}^{3} & S_{4}^{b}=L\left(\xi_{b}^{3}-\xi_{b}^{2}\right) \end{array},$$

where $\xi_{b}=x/L$, *L* is the length of the element and $e_{b}$ is the node coordinate vector composed of node positions and slope coordinates, which are absolute variables defined in the global coordinate system. For node *A*, the node coordinate vector is defined as follows:

(5) $$e_{b}^{A}={\left[\begin{array}{cc} {\left(r_{b}^{A}\right)}^{T} & {\left(\frac{\partial r_{b}^{A}}{\partial x}\right)}^{T} \end{array}\right]}^{T}={\left[\begin{array}{cccccc} r_{bx}^{A} & r_{by}^{A} & r_{bz}^{A} & \frac{\partial r_{bx}^{A}}{\partial x} & \frac{\partial r_{by}^{A}}{\partial x} & \frac{\partial r_{bz}^{A}}{\partial x} \end{array}\right]}^{T}.$$

As shown in Figure 1, the node coordinate vector of the element can be represented as follows:

(6) $$e_{b}={\left[\begin{array}{cc} {\left(e_{b}^{A}\right)}^{T} & {\left(e_{b}^{B}\right)}^{T} \end{array}\right]}^{T}.$$

The strain energy of the reduced beam element is composed of two parts: axial deformation and bending deformation, and the specific expressions are as follows:

(7) $$U^{b}=\frac{1}{2}\int_{0}^{l}\left[E_{b}A^{b}{\left(\varepsilon^{b}\right)}^{2}+E_{b}I^{b}{\left(\kappa^{b}\right)}^{2}\right]dx,$$

where $E_{b}$ is the elastic modulus of the cable, $A^{b}$ is the cross-sectional area of the cable, and $I^{b}$ is the moment of inertia of the cable. The axial strain of cable $\varepsilon^{b}$ and curvature $\kappa^{b}$ can be expressed as follows:

(8) $$\varepsilon^{b}=\frac{1}{2}\left[{\left(r_{b,x}\right)}^{T}r_{b,x}-1\right],$$

(9) $$\kappa^{b}=\frac{r_{b,x}\times r_{b,xx}}{{\left|r_{b,x}\right|}^{3}}.$$

where $r_{b,x}$ is represented as $r_{b}$ taking a partial derivative of *x* and $r_{b,xx}$ is represented as $r_{b}$ taking the double partial derivative of *x*; this representation is universal throughout the entire text.

The elastic force of the reduced beam element can be expressed as follows:

(10) $$Q_{e}^{b}=\frac{\partial U^{b}}{\partial e}.$$

### 2.2. Thin-Film Elements

**Figure 2:**
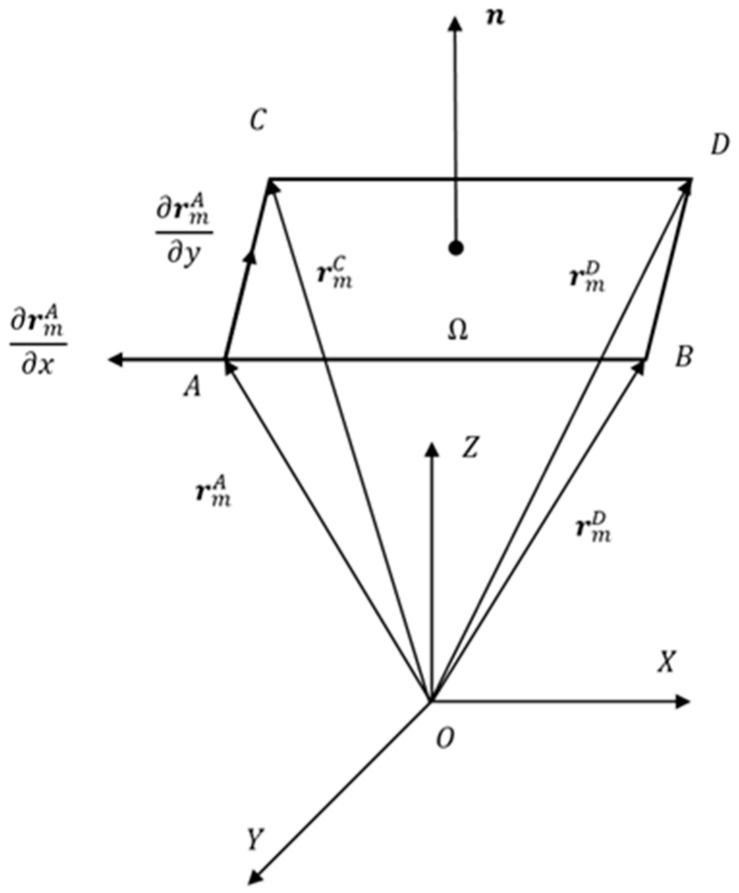
Schematic diagram of thin-film element.

Thin-plate elements can be used to describe the cross-sectional deformation of thin plates, while for thin films and non-uniform elements, their thickness is much smaller than the length in the other two directions, and their compression deformation in the thickness direction is generally not considered, so the slope vector in the thickness direction can be ignored. Taking the film as an example, the film element is divided into $k_{1}\times k_{2}$ elements, each containing four nodes and 36 degrees of freedom. Any element within the film is taken and a global coordinate system is established, as shown in Figure 2.

Among them, $r_{m}^{A}$, $r_{m}^{B}$, $r_{m}^{C}$, and $r_{m}^{D}$ are the loss of diameter of nodes *A*, *B*, *C*, and *D* within the thin film element relative to the global coordinate origin, respectively. ***n*** is the normal vector of the middle plane of the thin film. Assuming that the normal vector perpendicular to the middle plane of the element before deformation remains perpendicular to the middle plane after deformation, the normal vector ***n*** can be expressed as follows:

(11) $$\begin{array}{l} n=r_{m,x}\times r_{m,y},\\ \left\|n\right\|=\sqrt{{\left(r_{m,x}\times r_{m,y}\right)}^{T}\left(r_{m,x}\times r_{m,y}\right)}. \end{array}$$

The shape function and node vector coordinates of the thin-film element can be represented as follows:

(12) $$r_{m}=S^{m}\left(x,y\right)e_{m},$$

where $S^{m}\left(x,y\right)$ is a thin-film element shape function matrix represented by spatial coordinates *x* and *y*, which can be expressed as follows:

(13) $$S^{m}\left(x,y\right)=\left[\begin{array}{cccc} S_{1}^{m}I_{3} & S_{2}^{m}I_{3} & \cdots & S_{12}^{m}I_{3} \end{array}\right],$$

where the elements of the shape function can be expressed as follows:

(14) $$\begin{array}{l} S_{1}^{m}=-\left(\xi_{m}-1\right)\left(\eta_{m}-1\right)\left(2\xi_{m}^{2}+2\eta_{m}^{2}-\eta_{m}-\xi_{m}-1\right),\\ S_{2}^{m}=-b\xi_{m}{\left(\xi_{m}-1\right)}^{2}\left(\eta_{m}-1\right),\\ S_{3}^{m}=-b\eta_{m}\left(\xi_{m}-1\right){\left(\eta_{m}-1\right)}^{2},\\ S_{4}^{m}=\xi_{m}\left(\eta_{m}-1\right)\left(2\xi_{m}^{2}+2\eta_{m}^{2}-\eta_{m}-3\xi_{m}\right),\\ S_{5}^{m}=-b\xi_{m}^{2}\left(\xi_{m}-1\right)\left(\eta_{m}-1\right),\\ S_{6}^{m}=-c\xi_{m}\eta_{m}{\left(\eta_{m}-1\right)}^{2},\\ S_{7}^{m}=\eta_{m}\left(\xi_{m}-1\right)\left(2\xi_{m}^{2}+2\eta_{m}^{2}-3\eta_{m}-\xi_{m}\right),\\ S_{8}^{m}=b\xi_{m}\eta_{m}{\left(\xi_{m}-1\right)}^{2},\\ S_{9}^{m}=b\eta_{m}^{2}\left(\xi_{m}-1\right)\left(\eta_{m}-1\right),\\ S_{10}^{m}=-\xi_{m}\eta_{m}\left(1-3\xi_{m}-3\eta_{m}+2\xi_{m}^{2}+2\eta_{m}^{2}\right),\\ S_{11}^{m}=b\xi_{m}^{2}\eta_{m}{\left(\xi_{m}-1\right)}^{2},\\ S_{12}^{m}=c\xi_{m}\eta_{m}^{2}\left(\eta_{m}-1\right), \end{array}$$

where $\xi_{m}=k_{1}/b$, $\eta_{m}=k_{2}/c$, *b*, and *c* are the length and width of the element, respectively.

For thin-film elements, the node coordinate vectors of node *A* and thin-film elements can be represented as follows:

(15) $$\begin{array}{cc} e_{m}^{A} & ={\left[\begin{array}{cccc} {\left(r_{m}^{A}\right)}^{T} & {\left(\frac{\partial r_{m}^{A}}{\partial x}\right)}^{T} & {\left(\frac{\partial r_{m}^{A}}{\partial y}\right)}^{T} & {\left(\frac{\partial r_{m}^{A}}{\partial z}\right)}^{T} \end{array}\right]}^{T}\\ & =\left[\begin{array}{cccccc} r_{mx}^{A} & r_{my}^{A} & r_{mz}^{A} & \frac{\partial r_{mx}^{A}}{\partial x} & \frac{\partial r_{mx}^{A}}{\partial y} & \frac{\partial r_{mx}^{A}}{\partial z} \end{array}\right.{\left.\begin{array}{cccccc} \frac{\partial r_{my}^{A}}{\partial x} & \frac{\partial r_{my}^{A}}{\partial y} & \frac{\partial r_{my}^{A}}{\partial z} & \frac{\partial r_{mz}^{A}}{\partial x} & \frac{\partial r_{mz}^{A}}{\partial y} & \frac{\partial r_{mz}^{A}}{\partial z} \end{array}\right]}^{T}, \end{array}$$

(16) $$e_{m}={\left[\begin{array}{cccc} {\left(e_{m}^{A}\right)}^{T} & {\left(e_{m}^{B}\right)}^{T} & {\left(e_{m}^{C}\right)}^{T} & {\left(e_{m}^{D}\right)}^{T} \end{array}\right]}^{T}.$$

The elastic potential energy in vibration can be expressed as follows:

(17) $$U_{1}^{m}=\iint_{\Omega }\left\{\frac{E_{m}t_{m}}{24\left(1-\nu_{m}^{2}\right)}\left[{\left(\kappa_{xx}^{m}\right)}^{2}+{\left(\kappa_{yy}^{m}\right)}^{2}\right]\right.\left.+\frac{\nu_{m}E_{m}t_{m}}{12\left(1-\nu_{m}^{2}\right)}\kappa_{xx}^{m}\kappa_{yy}^{m}+\frac{E_{m}t_{m}}{12\left(1-\nu_{m}^{2}\right)}\kappa_{xy}^{2}\right\}dxdy.$$

The tensile strain potential energy is as follows:

(18) $$U_{2}^{m}=\frac{E^{m}t_{m}}{2\left(1-\nu_{m}^{2}\right)}\iint_{\Omega }\left[{\left(\varepsilon_{x}^{m}\right)}^{2}+{\left(\varepsilon_{y}^{m}\right)}^{2}+2\nu_{m}{\left(\varepsilon_{x}^{m}\varepsilon_{y}^{m}\right)}^{2}+\frac{1-\nu_{m}}{2}{\left(\varepsilon_{xy}^{m}\right)}^{2}\right]dxdy,$$

where $E_{m}$, $\nu_{m}$, and $t_{m}$ are the elastic modulus, Poisson’s ratio, and thickness of the thin film, respectively. According to continuous medium mechanics, the elastic force vector of an element is the partial derivative of the element’s strain energy with respect to the generalized coordinates. Therefore, the elastic force vector of the thin-film element can be represented as follows:

(19) $$Q_{e}^{m}=\frac{\partial U_{1}^{m}}{\partial e^{m}}+\frac{\partial U_{2}^{m}}{\partial e^{m}}=Q_{1}^{m}+Q_{2}^{m}.$$

The strain and curvature vector can be expressed as follows:

(20) $$\begin{array}{l} \varepsilon_{ij}^{m}=\frac{1}{2}\left(e_{m}^{T}S_{,i}^{T}S_{,j}^{T}e_{m}-\delta_{ij}\right),\\ \kappa_{ij}^{m}=\frac{r_{m,ij}^{T}n}{{\left\|n\right\|}^{3}}. \end{array}$$

### 2.3. Element Quality Matrix and External Force Matrix

In the ANCF, the element kinetic energy in global coordinates can be expressed as follows:

(21) $$F_{e}=\frac{1}{2}\int_{V_{e}}\rho {\dot{r}}^{T}\dot{r}dV_{e},$$

where ρ is the density of the element, and $V_{e}$ is the volume of the element. Substituting Equation (10) into Equation (19) gives the following:

(22) $$F_{e}=\frac{1}{2}{\dot{e}}^{T}M_{e}\dot{e}.$$

The element quality matrix can be expressed as follows:

(23) $$M_{e}=\int_{V_{e}}\rho S^{T}SdV_{e}.$$

Taking thin-film elements as an example, when an external force ***T*** acts on a thin-film element, the virtual work of that force can be expressed as follows:

(24) $$\delta W=T^{T}\delta r=T^{T}S\delta e=F_{e}^{T}\delta e.$$

At this time, the external force matrix of the thin-film element can be represented as follows:

(25) $$F_{e}^{T}=S{\left(x,y\right)}^{T}T.$$

### 2.4. Element Rigid Body Motion

Taking the thin-film element as an example, the element node coordinate vector of the thin-film element during motion can be represented as follows:

(26) $$\begin{array}{l} e_{m}^{A}={\left[\begin{array}{ccccccccc} r_{x}^{A} & r_{y}^{A} & r_{z}^{A} & N_{11} & N_{21} & N_{31} & N_{12} & N_{22} & N_{32} \end{array}\right]}^{T},\\ e_{m}^{B}=\left[\begin{array}{ccccccccc} r_{x}^{A}+N_{11}b & r_{y}^{A}+N_{21}b & r_{z}^{A}+N_{31}b & N_{11} & N_{21} & N_{31} & N_{12} & N_{22} & N_{32} \end{array}\right],\\ e_{m}^{C}=\left[\begin{array}{ccccccccc} r_{x}^{A}+N_{11}b+N_{12}c & r_{y}^{A}+N_{21}b+N_{22}c & r_{z}^{A}+N_{31}b+N_{32}c & N_{11} & N_{21} & N_{31} & N_{12} & N_{22} & N_{32} \end{array}\right],\\ e_{m}^{D}=\left[\begin{array}{ccccccccc} r_{x}^{A}+N_{12}c & r_{y}^{A}+N_{22}c & r_{z}^{A}+N_{32}c & N_{11} & N_{21} & N_{31} & N_{12} & N_{22} & N_{32} \end{array}\right], \end{array}$$

where $N_{ij}$ represents the i-th row and j-th column of the 3×3 rigid body rotation matrix. $r_{x}^{A}$, $r_{y}^{A}$, and $r_{z}^{A}$ are the global translation position vectors of the middle surface of the thin-film element, respectively. Euler angles ϕ, η, and ψ represent the rotation angles of the thin-film element about the coordinate axes. Therefore, the rotation matrix ***N*** can be expressed as follows:

(27) $$N=\left[\begin{array}{ccc} \cos\psi \cos\phi -\cos\eta \sin\phi \sin\psi & -\sin\psi \cos\phi -\cos\eta \sin\phi \cos\psi & 0\\ \cos\psi \sin\phi +\cos\eta \cos\phi \sin\psi & -\sin\psi \sin\phi +\cos\eta \cos\phi \cos\psi & 0\\ \sin\eta \sin\psi & \sin\eta \cos\psi & 0 \end{array}\right].$$

## 3. Dynamic Equations of Thin-Film Structures Considering Wrinkles

### 3.1. Wrinkling Deformation under Pre-Tension

**Figure 3:**
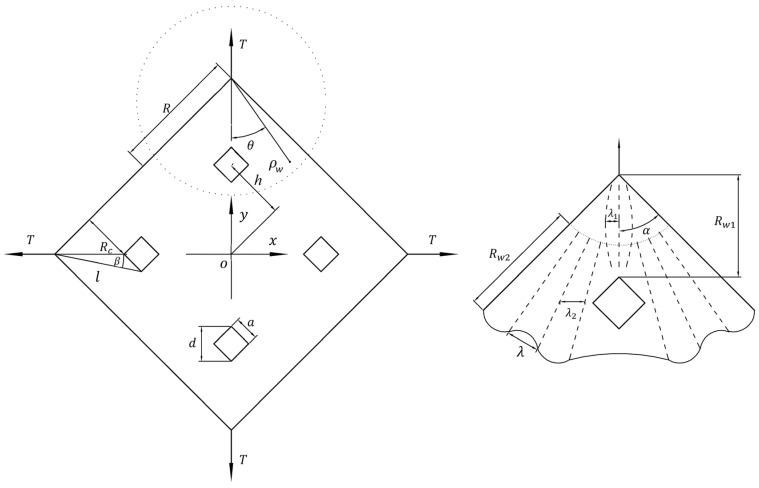
Schematic diagram of film and wrinkle parameters.

The schematic diagram of the non-uniform film structure is shown in Figure 3. We found through experimental research that the wrinkling morphology of non-uniform films is related to the size of non-uniform elements; we discussed this phenomenon in detail in reference [37]. For the thin-film structures of non-uniform elements with periodic distribution on the diagonal, when the size of the non-uniform elements a>2λ and a≤2λ, the wrinkling models of non-thin-films under uniform tension are as follows [37]:

(28) $$\begin{array}{l} w_{1}=A_{11}\sin\left(\frac{\pi \rho_{w}}{R_{w1}}\right)\sin\left(\frac{\pi \theta }{\lambda }\right)+A_{12}\sin\left[\frac{\pi \left(\rho_{w}-l+\frac{R_{w2}}{2}\right)}{R_{w2}}\right]\sin\left[\frac{\pi \left(\theta -\beta \right)}{\lambda }\right],\\ A_{11}=e^{-{\left(3t^{m}\left|x\right|\right)}^{2}}A\left(\frac{R_{w1}}{2},0\right),A_{12}=e^{-{\left[3t^{m}(\left|x\right|-\frac{d+\lambda_{2}}{2})\right]}^{2}}A\left(l,\beta \right), \end{array}$$

(29) $$\begin{array}{l} w_{2}=A_{21}\sin\left(\frac{\pi \rho_{w}}{R_{w}}\right)\cos\left(\frac{\pi \theta }{\lambda }\right)+A_{22}\sin\left[\frac{\pi \left(\rho_{w}-l\right)}{R_{w2}}\right]\cos\left[\frac{\pi \left(\theta -\beta \right)}{\lambda }\right],\\ A_{21}=e^{-{\left[3t_{m}\left(\left|x\right|-\frac{\lambda_{2}}{2}\right)\right]}^{2}}A\left(\frac{R_{w1}}{2},0\right),A_{22}=e^{-{\left[3t_{m}(\left|x\right|-\frac{2\lambda_{1}+\lambda_{2}}{2})\right]}^{2}}A\left(l,\beta \right), \end{array}$$

where $R_{w}$ is the folding radius, which can be expressed as follows:

(30) $$\begin{array}{l} R_{w}=\exp\left(-\nu_{m}\right)R,\\ R_{w1}=R_{c}/\sin\alpha ,\\ R_{w2}=R_{w}-R_{w1}, \end{array}$$

where $A_{ij}$ is the amplitude of wrinkles, which can be expressed as follows:

(31) $$A\left(\rho_{w},\theta \right)=2\lambda \sqrt{\left(-\frac{4T\nu_{m}\cos\theta }{\left(\pi +2\right)\pi^{2}E_{m}\rho_{w}t_{m}}-\int \frac{4T\cos\theta }{\left(\pi +2\right)\pi^{2}E_{m}\rho_{w}t_{m}y}dy\right)},$$

where λ is wrinkle radius can be expressed as follows:

(32) $$\begin{array}{l} \lambda ={\left[\frac{E_{m}\rho_{w}\pi^{2}\left(\pi +2\right)t_{m}^{3}R_{w}^{2}}{48T\left(1-\nu_{m}^{2}\right)\cos\theta }\right]}^{\frac{1}{4}},\\ \lambda_{1}=\left(\frac{R_{c}}{\sin\alpha }+\frac{d}{2}\right)\tan\left(\frac{\lambda }{R_{w}}\right),\\ \lambda_{2}=\left(\frac{R_{c}}{\sin\alpha }+\frac{d}{2}\right)\tan\left(\frac{\alpha }{n_{w}}+\beta \right)-\frac{d}{2}, \end{array}$$

where $n_{w}$ is the number of wrinkles.

**Figure 4:**
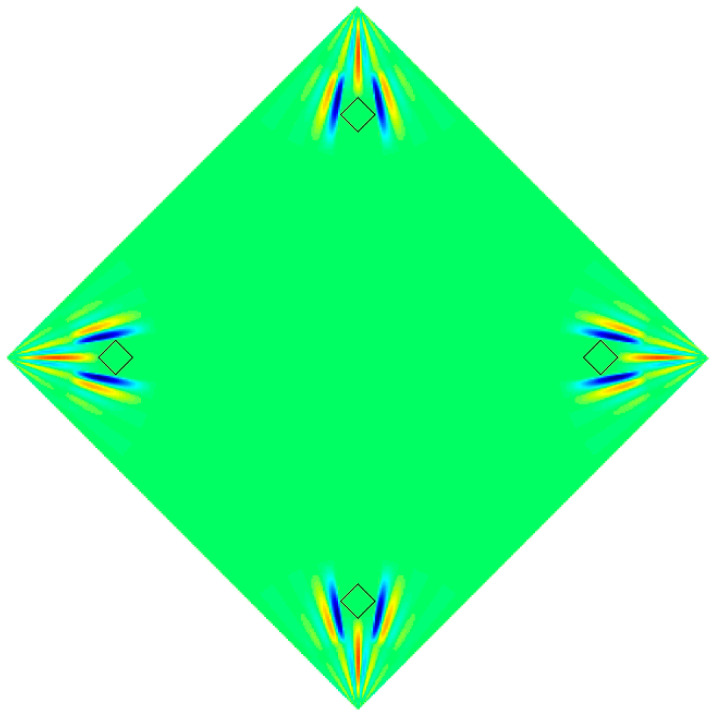
Schematic diagram of wrinkle deformation.

According to Equations (28)–(32), the wrinkling deformation of non-uniform thin films under uniform tension can be obtained, the wrinkled prediction results are shown in Figure 4, where reddish and bluish colors indicate the fold deformation of the film in the positive and negative directions, respectively. Finally, combined with Equations (26) and (27), it can be substituted as the initial form into the absolute node coordinate method.

### 3.2. The Handling of Constraint Conditions

There are motion constraints between thin films and cables, as well as between thin films and non-uniform components. The coordinates of two different element nodes at the end of the cable and the corner vertex of the film have the same spatial node displacement and different cross-sectional slopes. The coordinates of element nodes in contact with the film and non-uniform elements have the same spatial node displacement and cross-sectional slope. For the convenience of handling constraint conditions, the elastic force of the element can be expressed as the product of the stiffness matrix and node coordinates:

(33) $$Q_{e}=K_{e}e.$$

Taking the connection between the cable and the thin film as an example, in order to illustrate the constraints between two nodes, each node only takes three vectors representing displacement for derivation, and assuming that they have the same y-direction displacement, the constrained equation is as follows:

(34) $$\left[\begin{array}{cccccc} k_{11} & 0 & k_{13} & k_{14} & k_{15}+k_{12} & k_{16}\\ 0 & 1 & 0 & 0 & -1 & 0\\ k_{31} & 0 & k_{33} & k_{34} & k_{35}+k_{32} & k_{36}\\ k_{41} & 0 & k_{43} & k_{44} & k_{45}+k_{42} & k_{46}\\ k_{51}+k_{12} & -1 & k_{53}+k_{32} & k_{54}+k_{42} & k_{55}+2k_{52}+k_{22}+1 & k_{56}+k_{62}\\ k_{61} & 0 & k_{63} & k_{64} & k_{65}+k_{62} & k_{66} \end{array}\right]\left[\begin{array}{c} e_{1}\\ e_{2}\\ e_{3}\\ e_{4}\\ e_{5}\\ e_{6} \end{array}\right]=\left[\begin{array}{c} f_{1}\\ 0\\ f_{3}\\ f_{4}\\ f_{5}-f_{2}\\ f_{6} \end{array}\right].$$

The constraint conditions between thin films and non-uniform elements can be processed using the above methods for computer programming in the future.

### 3.3. Dynamic Equations

The elements are connected at the nodes, and the global stiffness matrix, global external force matrix, and global mass matrix of the thin-film structure can be expressed by using the Boolean matrix ***H*** between the element node coordinates and the generalized coordinates:

(35) $$\begin{array}{l} K=\sum_{n}H^{T}K_{e}e,\\ F=\sum_{n}H^{T}F_{e},\\ M=\sum_{n}H^{T}M_{e}H. \end{array}$$

According to the Lagrange equation, the dynamic equation of the thin-film structure can be obtained as follows:

(36) $$M\ddot{e}+Ke=F.$$

In Equations (35) and (36), $\ddot{e}$ is the acceleration vector and ***M*** is a constant matrix used to describe the mass distribution inside the structure, which can be calculated and stored in advance.

Due to the fluid structure coupling effect, the mass matrix of the thin film structure will increase when the thin film vibrates in the air. Therefore, the Minami additional mass ratio formula was used to modify the dynamic equation of the thin film. The modified dynamic equation is as follows [38]:

(37) $$\left(1+\alpha_{m}\right)M\ddot{e}+Ke=F,$$

where $\alpha_{m}$ is the additional mass ratio representing the ratio of air mass to the mass of the vibrating film in the air, regardless of vibration frequency and amplitude. The equivalent air layer height corresponding to the uniformly distributed additional mass in the film structure is approximately 68% of the length of the film structure, which can be expressed as follows:

(38) $$\alpha_{m}=0.68\rho_{a}l_{m}/m_{m},$$

where $\rho_{a}$ is the mass density of the air, $m_{m}$ is the mass of the thin-film structure, and $l_{m}$ is the length of the thin film.

**Figure 5:**
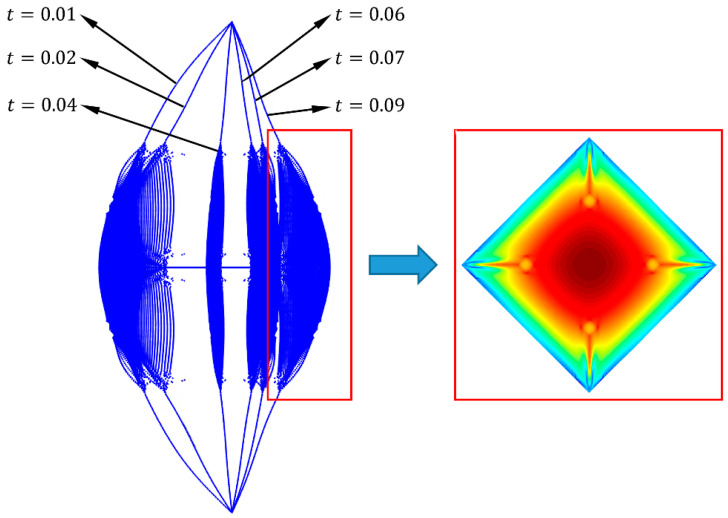
Node deformation of the thin-film system at different times.

When the film center is subjected to an impact force of 0.2 N, a dynamic model is established for the film structure with a rigid element size of 20×20×1 mm, a film size of 300×300×0.025 mm, and the cable is a steel cable with a diameter of 1 mm and a length of 100 mm. The parameters of the film and cable remain unchanged in the following text. The Newmark method was used to solve the dynamic equation [33], and the node deformation of the film system at different times is obtained as shown in Figure 5. The red-boxed portion of Figure 5 represents the displacement cloud diagram in the direction normal to the film, where reddish and bluish colors indicate the displacement of the film in the positive and negative directions, respectively. From the red box on the right side of Figure 5, it can be seen that the non-uniform film exhibited wrinkles during vibration.

## 4. Experimental Verification and Vibration Characteristic Analysis

### 4.1. Experimental Measurement System

**Figure 6:**
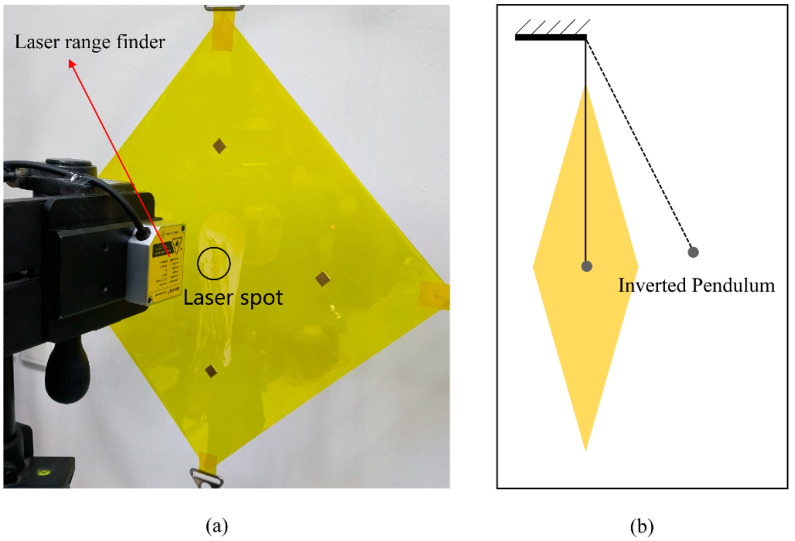
Measurement system for the thin-film impact vibration experiment: (**a**) is a non-uniform thin-film structure and a laser rangefinder; (**b**) is the method of applying the impact force.

In order to verify the effectiveness of the absolute node coordinate method used in this article, an experimental system was designed as shown in Figure 6. The laser rangefinder had a resolution of 0.002 mm, a sampling frequency of 500 Hz, and an impact effect was applied using a small, inverted pendulum.

### 4.2. The Influence of Non-Uniform Element Position on the Vibration Characteristics of Thin Films

**Figure 7:**
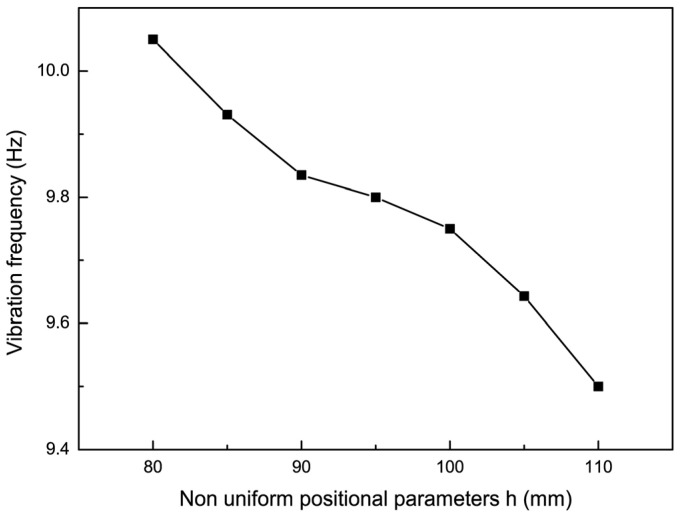
The trend of vibration frequency changing with non-uniform element position parameters.

**Figure 8:**
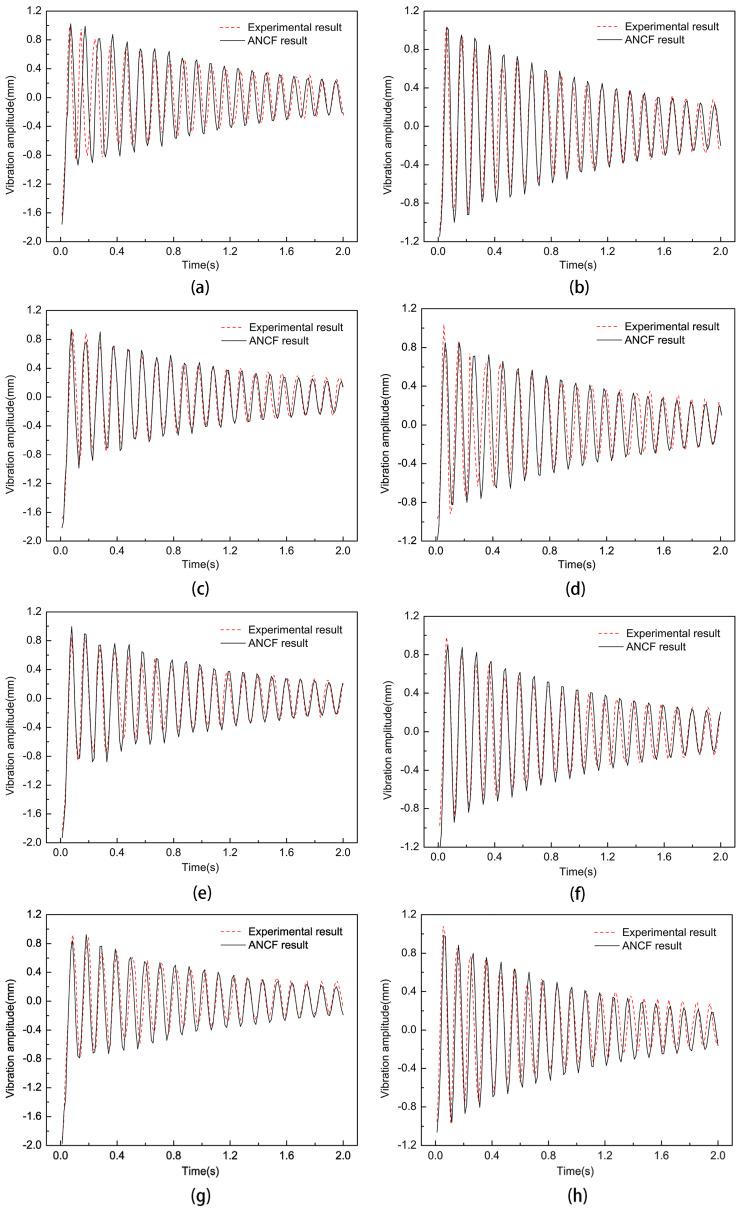
Vibration curves of thin-film structures with non-uniform elements at different positions: (**a**,**c**,**e**,**g**) are the vibration curves of the center point of the thin film when the non-uniform element position parameter h is 80 mm, 90 mm, 100 mm, and 110 mm, respectively; (**b**,**d**,**f**,**h**) are the vibration curves of the center point of the film when the non-uniform element position parameter h is 80 mm, 90 mm, 100 mm, and 110 mm, respectively.

The effect of non-uniform cell position on the dynamic characteristics of the thin-film structure was investigated such that the size parameter of the non-uniform element was 10 mm, the pre-tension was 15 N, and an impact force of 0.2 N was applied. The experimental results of the trend of the vibration frequency variation with the non-uniform element position parameter when the non-uniform unit position parameter was 80, 85, 90, 95, 100, 105, and 110 are shown in Figure 7. Then, the dynamic analysis of the film structure with non-uniform cell position parameters h of 80 mm, 90 mm, 100 mm, and 110 mm was carried out to extract the vibration curves of the film center and non-uniform cells, which were compared with the experimental results, as shown in Figure 8.

From Figure 7 and Figure 8, it can be seen that as the size parameter h of the film gradually increased, the vibration frequency of the film structure gradually increased. During vibration, the frequency of vibration between the transients of the thin-film structure changed slightly under the influence of the introduction of non-uniform elements as the positional parameter of the non-uniform elements, h, gradually increased, compared to when the position parameter of the non-uniform element was 80 mm; then, the vibration frequencies of non-uniform component position parameters decreased by 1.6%, 2.5%, and 5.1% at 90, 100, and 110, respectively. This was because when the position of the non-uniform element changed, the stress distribution of the thin-film structure changed accordingly, but the total mass of the thin-film structure remained unchanged. Therefore, the vibration frequency and amplitude of the thin-film structure underwent slight changes under the influence of the changed stress distribution.

**Figure 9:**
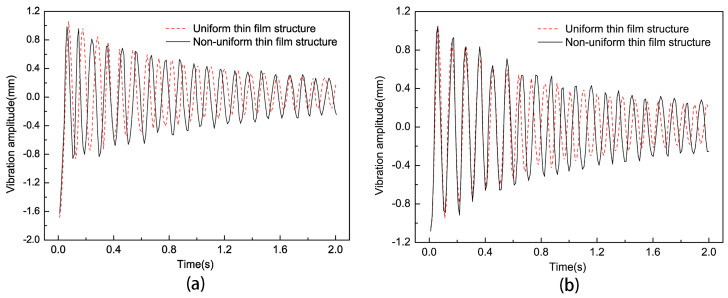
Vibration curves of uniform thin films and non-uniform thin films: (**a**,**b**) are the vibration curves of the center of the thin film and the center point of the non-uniform element, respectively.

To study the influence of non-uniform elements on the vibration characteristics of thin-film structures, the size and position parameters of non-uniform elements were kept constant. We compared the vibration curves of the center point of the non-uniform and uniform thin-film structures through experiments and compared the vibration curves of non-uniform element centers and uniform thin films at corresponding positions; the results are shown in Figure 9. According to the experimental results, the maximum and minimum amplitude differences of the last vibration cycle of the uniform film and non-uniform film at the center point of the film were 0.366 mm and 0.513 mm, respectively; The maximum and minimum amplitude differences of the last vibration cycle corresponding to the center point of the non-uniform element were 0.409 mm and 0.539 mm, respectively. It can be seen that non-uniform elements had a significant impact on the amplitude of the thin-film vibration. At the same time, the vibration frequency decreased by 6.82% compared to the non-uniform thin-film structure.

### 4.3. The Influence of Non-Uniform Element Size on the Vibration Characteristics of Thin Films

**Figure 10:**
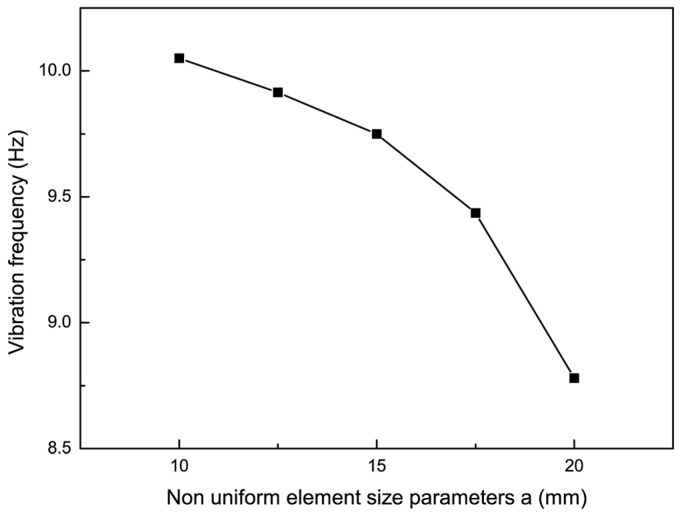
The trend of vibration frequency changing with non-uniform element size parameters.

**Figure 11:**
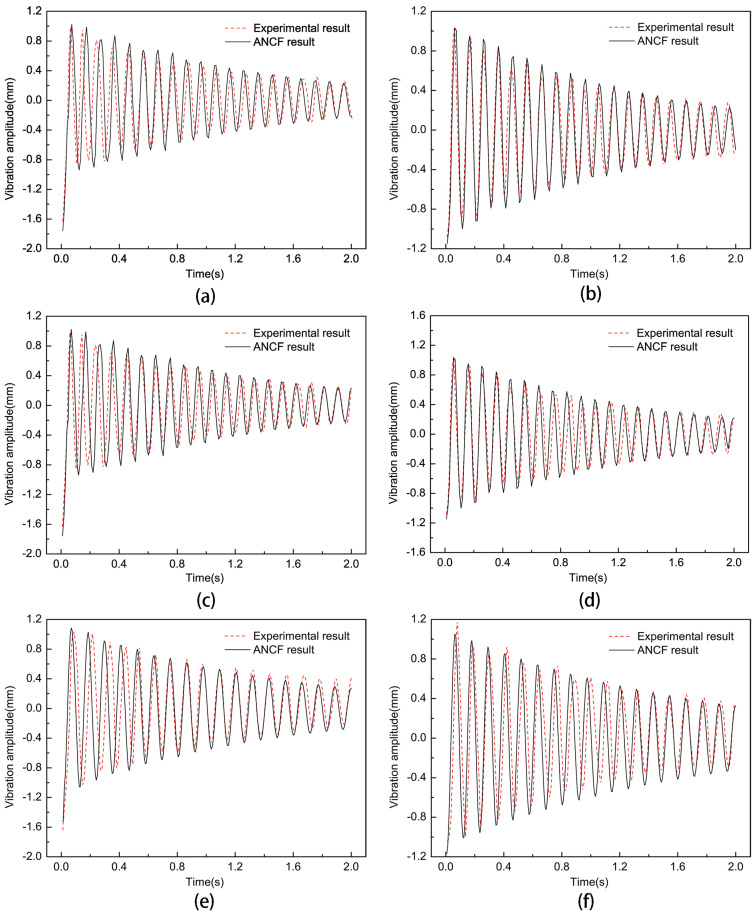
Vibration curves of non-uniform elements at different size parameters: (**a**,**c**,**e**) are the vibration curves of the center point of the thin film when the non-uniform element size parameter h is 10 mm, 15 mm, and 20 mm, respectively; (**b**,**d**,**f**) are the vibration curves of the center point of the film when the non-uniform element size parameter h is 10 mm, 15 mm, and 20 mm, respectively.

To investigate the effect of non-uniform cell size on the vibration characteristics of the film, the non-uniform element position parameter h was made to be 80 mm, the pre-tension was 15 N, and an impact force of 0.2 N was applied. The experimental results of the trend of the vibration frequency with the non-uniform element size parameter are shown in Figure 10 when the non-uniform element size parameters were 10, 12.5, 15, 17.5, and 20. Then, the dynamic analysis of the thin-film structure containing non-uniform elements of different sizes was carried out, respectively, and the vibration curves of the thin film and the center of the non-uniform unit were extracted when the non-uniform unit size parameters were 10, 15, and 20, respectively, and were compared with the experimental results, as shown in Figure 11. By comparing the analytical and experimental results, it can be seen that the vibration amplitude of the non-uniform film structure increased slowly with the increase of the non-uniform film size parameter a. Meanwhile, the vibration frequency of the film decreased by 4.88% and 12.2% when the non-uniform element size parameters were 15 and 20 mm, respectively.

### 4.4. The Influence of Pre-Tension on the Vibration Characteristics of Non-Uniform Thin Films

**Figure 12:**
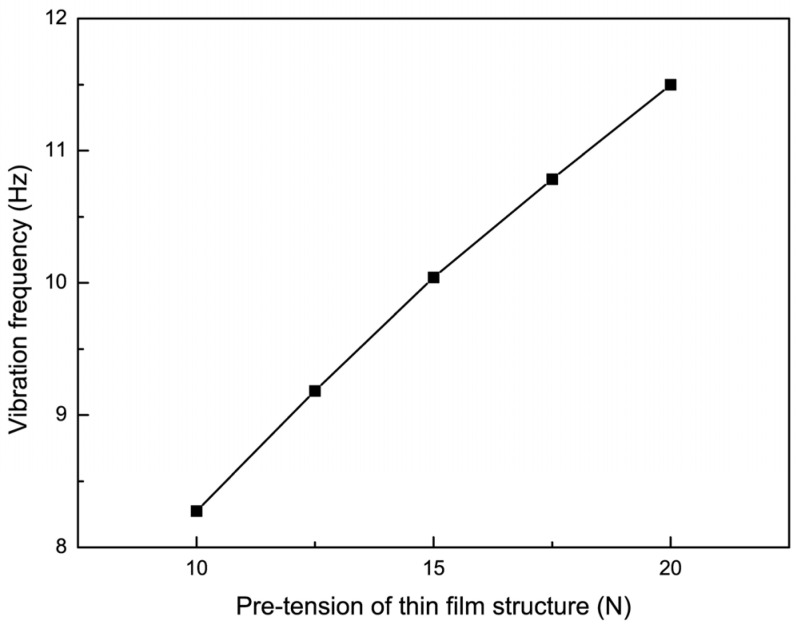
The trend of vibration frequency changing with pre-tension of thin film.

**Figure 13:**
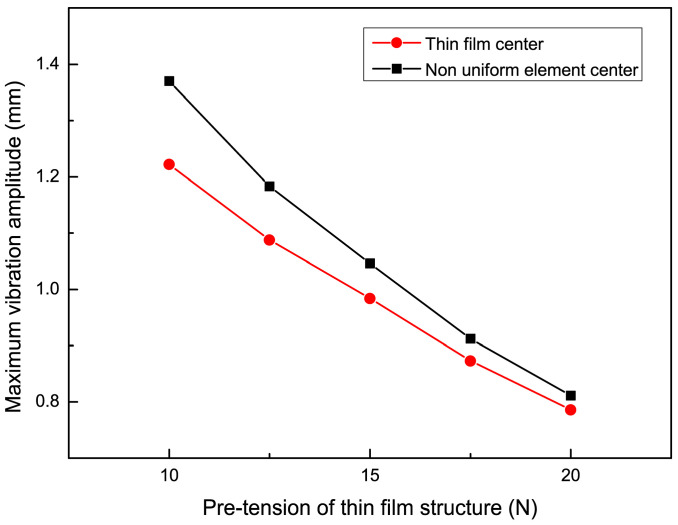
The trend of maximum vibration amplitude changing with pre-tension of thin film.

**Figure 14:**
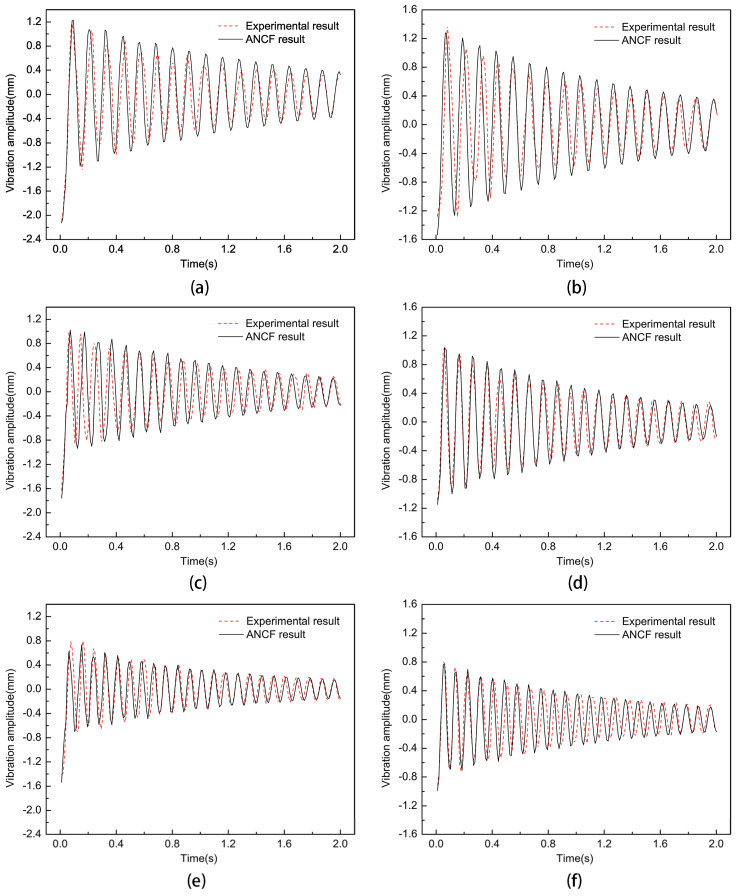
Vibration curves of non-uniform thin films under different pre-tensions: (**a**,**c**,**e**) are the vibration curves of the center point of the film under tensions of 10 N, 15 N, and 20 N, respectively; (**b**,**d**,**f**) are the vibration curves of the center point of the film under tensions of 10 N, 15 mm, and 20 N, respectively.

To study the effect of non-uniform cell size on the vibration characteristics of the film, an impact force of 0.2 N was applied such that the position parameter h was 80 mm, and the cell size parameter was 10 mm. When the applied pre-tension was 10 N, 12.5 N, 15 N, 17.5 N, and 20 N, the experimental results of the vibration frequency with the trend of the non-uniform film size parameter are shown in Figure 12, and the experimental results of the maximum vibration amplitude with the trend of the non-uniform cell size parameter are shown in Figure 13. Then, the dynamic analysis of the film structure was carried out and the vibration curves of the film and the center of the non-uniform unit at pre-tensions of 10 N, 15 N, and 20 N were extracted and compared with the experimental results, which are shown in Figure 14. It can be concluded that when the pre-tension was set to 15 N and 20 N, the vibration frequency increased by 20.58% and 38.24%, respectively, compared to the non-uniform film with a pre-tension of 10 N. Meanwhile, the vibration amplitude of the non-uniform film structure decreased significantly when the pre-tension was increased, and the maximum vibration amplitude at the center of the film decreased by 14.5% and 35.7%. The maximum amplitude at the center of the non-uniform element decreased by 23.6% and 40.8%, respectively, when the pre-tension was up to 15 N and 20 N, as compared to non-uniform films with a pre-tension of 10 N. Therefore, the selection of effective pre-tensioning is essential to improve the performance of the films.

## 5. Conclusions

This article establishes a dynamic model of non-uniform tensioned thin-film structures based on the absolute node coordinate method, taking into account the wrinkling deformation of the thin films under pre-tension and the additional air mass when the thin film vibrates in the air. The effectiveness of the model was verified through experiments. The influence of non-uniform element position parameters, size parameters, and film pre-tension on the vibration characteristics of thin-film structures was studied. According to the analysis results, the position and size of non-uniform elements had relatively little effect on the vibration frequency of the thin film. When the position parameter of the thin film increased from 80 mm to 90 mm, 100 mm, and 110 mm, the vibration frequency of the thin film decreased by 1.6%, 2.5%, and 5.1%, respectively. When the size parameter of the film increased from 10 mm to 15 mm and 20 mm, the vibration frequency of the film decreased by 4.8% and 12.2%, respectively. The introduction of non-uniform elements had a significant impact on the vibration amplitude of the thin film, but a relatively small impact on the vibration frequency of the thin film. When the position parameters, size parameters, and pre-tension of the non-uniform elements were 80 mm, 15 mm, and 15 N, respectively. Compared to the uniform thin film structure, the introduction of non-uniform elements increased the vibration frequency of the thin film by 7%, and the vibration amplitudes at the center of the thin film and the center of the non-uniform element increased by 40.2% and 47.3%, respectively. The pre-tension of the film had a significant impact on the vibration amplitude and frequency of the non-uniform films. When the position and size parameters of the non-uniform element were 80 mm and 15 mm, respectively, and the pre-tension of the film increased from 10 N to 15 N and 20 N, the vibration frequency of the film increased by 20.58% and 38.24%, respectively. The maximum vibration amplitude of the film at the center point decreased by 14.5% and 35.7%, respectively, and the maximum vibration amplitude at the center point of the non-uniform element decreased by 23.6% and 40.8%, respectively. Therefore, when designing thin-film structures, it is necessary to choose reasonable non-uniform element sizes and thin-film pre-tensions to reduce the impact on the performance of the thin film structure.

## Data Availability

The original contributions presented in the study are included in the article, further inquiries can be directed to the corresponding authors.
